# Gefitinib Synergizes with Irinotecan to Suppress Hepatocellular Carcinoma via Antagonizing Rad51-Mediated DNA-Repair

**DOI:** 10.1371/journal.pone.0146968

**Published:** 2016-01-11

**Authors:** Jinjin Shao, Zhifei Xu, Xueming Peng, Min Chen, Yuanrun Zhu, Li Xu, Hong Zhu, Bo Yang, Peihua Luo, Qiaojun He

**Affiliations:** Zhejiang Province Key Laboratory of Anti-Cancer Drug Research, Institute of Pharmacology and Toxicology, College of Pharmaceutical Sciences, Zhejiang University, Hangzhou 310058, China; Taipei Medicine University, TAIWAN

## Abstract

Chemotherapy is the only choice for most of the advanced hepatocellular carcinoma (HCC) patients, while few agents were available, making it an urgent need to develop new chemotherapy strategies. A phase II clinical trial suggested that the efficacy of irinotecan in HCC was limited due to dose-dependent toxicities. Here, we found that gefitinib exhibited synergistic activity in combination with SN-38, an active metabolite of irinotecan, in HCC cell lines. And the enhanced apoptosis induced by gefitinib plus SN-38 was a result from caspase pathway activation. Mechanistically, gefitinib dramatically promoted the ubiquitin–proteasome-dependent degradation of Rad51 protein, suppressed the DNA repair, gave rise to more DNA damages, and ultimately resulted in the synergism of these two agents. In addition, the increased antitumor efficacy of gefitinib combined with irinotecan was further validated in a HepG2 xenograft mice model. Taken together, our data demonstrated for the first time that the combination of irinotecan and gefitinib showed potential benefit in HCC, which suggests that Rad51 is a promising target and provides a rationale for clinical trials investigating the efficacy of the combination of topoisomerase I inhibitors and gefitinib in HCC.

## Introduction

Hepatocellular carcinoma (HCC) is a leading cause of cancer induced death[1] with high incidence and mortality worldwide[2]. Currently, the overall median survival for patients with advanced HCC was 6 months[3] due to limited treatment choice. The treatment options available for patients with HCC were surgery, early-stage radiofrequency ablation and chemotherapy[4]. Surgical treatment is curative for patients with early stage-HCC but not for advanced HCC patients[5]. Efficacy of radiofrequency ablation in advanced HCC was pretty low [6]. Thus chemotherapy is the only choice in most of the HCC patients.

For now, sorafenib is the standard systemic drug for advanced HCC [7]. Unfortunately, limited survival benefits due to drug resistance and intolerance restricted the use of sorafenib. Irinotecan has been used as first-line chemotherapy in patients with different solid tumors [8–11]. SN-38, the active metabolite of irinotecan has much more potent antitumor activity than irinotecan. Irinotecan or SN-38 treatment would lead to stabilization of cleavable topoisomerase I-DNA complex then result in DNA damages and give rise to G2/M cell-cycle arrest so that DNA damages could be repaired predominantly by Rad51 mediated homologous recombination(HR) repair pathway [12–14]. If the DNA damages were too severe to be repaired, apoptosis cascades would be activated thus irinotecan exhibited its antitumor activity. Unfortunately, phase II studies of irinotecan in HCC suggested that its activity is limited due to its dose-dependent toxicity [15, 16]. Therefore, it is a potential beneficial strategy to develop combination therapies to decrease its toxicity.

DNA repair systems have been proved to be as molecular targets of cancer therapy. Recent studies showed that inhibition of HR pathway could be a candidate for sensitization of chemotherapeutic drugs. And deletion of Rad51 gene would sensitize tumor cells to chemotherapeutic agents. Especially, gefitinib(GEFI) induced suppression of Rad51 was proved to be a novel strategy in cancer therapy [17]. Gefitinib is an orally active drug that inhibits EGFR-tyrosine kinase [18, 19]. Clinical study showed that gefitinib could prevent unresectable HCC development [20]. Interestingly, several studies showed that gefitinib enhanced the antitumor activity of cytotoxic drugs like irinotecan in gastric cancer via inhibiting EGF signals and IL-8 production [21, 22].

In our study, we confirmed the hypothesis that the combination of irinotecan with gefitinib might have the synergistic effects to HCC and demonstrated that synergistic effects was a result from accumulation of DNA damages caused by the defects of homologous recombination repair. The combination use of irinotecan and gefitinib might be a clinically effective strategy targeted to HCC.

## Materials and Methods

### Cell culture

The hepatocellular carcinoma cell lines, HepG2, Bel-7402 and SMMC-7721 were purchased from Cell Bank of China Science. Cells were cultured in DMEM /RPMI-1640 containing 10% fetal bovine serum and 0.1% antibiotics in a humidified atmosphere with 5% CO_2_ at 37°C.

### Reagents

Irinotecan and SN-38 were kindly provided by Dr. Wei Lu (East China Normal University). Gefitinib was purchased from the first Affiliated Hospital, Zhejiang University School of Medicine. Primary antibodies directed against β-actin, GAPDH, CHK1, CHK2, p53, ubiquitin and Rad51 and HRP-labelled secondary antibodies were purchased from Santa Cruz Biotechnology; Antibodies directed against cleaved PARP, cleaved caspase-3, p-CHK1, and p-CHK2 were obtained from Cell Signaling Technology. DMSO, propidium iodide (PI), sulforhodamine B (SRB), DAPI (4`, 6-diamidino-2-phenylindole), Tris-Base and Trichloroacetic acid were purchased from Sigma-Aldrich.

### Cell proliferation assay

Cell proliferation was assessed by the SRB colorimetric assay [23]. Cells in 96 well plates were exposed to drugs for 72h and then fixed with 10% Trichloroacetic acid and stained with SRB. SRB in the wells was dissolved in 10 mmol/L Tris-Base and measured at 510 nm using a multiwell spectrophotometer. The inhibition rate of cell proliferation was calculated for each well as (A510_control cells_-A510_treated cells_)/A510_control_ cells × 100%. And CI values are used to quantify drug synergism based on Chou-Talalay methodC[24–26]. A CI <0.90 indicates synergistic effects, a CI of 0.90 to 1.10 indicates an additive effect and a CI >1.10 indicates antagonistic effects. CI values at different ratio concentrations of gefitinib and SN-38 were calculated based on the proliferation assay results.

### Clonogenic assay

1,000 HepG2, Bel-7402 or SMMC-7721 cells were plated in a 6-well plate and treated with gefitinib and/or SN-38. Replace the medium with new medium containing 10% FBS every 2 to 3 days and add the drugs. After 10 days treatment, the cells were stained with a 0.1% crystal violet solution and photographed. Crystal violet was dissolved in 10% acetic acid and the absorbance values were determined at 595 nm. The clonogenic ability was calculated as follows: colony formation (% control) = [(A595_treated cells_-A595_blank_)/ (A595_control_-A595_blank_)] × 100%.

### Detection of apoptosis by Flow cytometry

Cells were treated with gefitinib and/or SN-38 for 36h and then harvested. Apoptosis rate was measured by an Annexin V-FITC/PI apoptosis Detection Kit. PI staining was also employed to assess apoptosis by analysis of the sub-G1 phase. Cells were harvested after treatment and fixed with 70% ethanol at -20°C. Cell were resuspended in 500 μl PBS containing 50 μg RNAase at 37°C for 30 min and then 5 μg PI was added to above mentioned solution in dark. Samples were then analyzed on a FACSCalibur cytometer (Becton Dickinson)

### Western blot analysis

Cells were harvested after drug treatment and resuspended in lysis buffer (50 mM Tris-HCl, 150 mM NaCl, 2 mM EDTA, 2 mM EGTA, 25 mM NaF, 25 mM β-Sodium Glycerophosphate, 0.3% NP-40, 0.3% Triton X-100, 0.25% Leupeptin, 0.1% PMSF, 0.1% NaVO_3_). Small pieces of tumor tissues were sonicated in lysis buffer on ice. Cell or tissue lysates were centrifuged at 13,200 rpm for 30 min at 4°C. Proteins were fractionated on Tris–glycine gels, and then transferred to nitrocellulose membrane and incubated with primary antibodies followed by HRP-labelled secondary antibodies. Protein level was then detected using ECL-plus kit and visualized on autoradiography film.

### Plasmids construction and transfection

The full-length Rad51 coding sequence was amplified from the HepG2 cDNA library using a pair of primers (Forward: CTTGGTACCGAGCTCGGATCCATGGCAATGCAGATGCAGCTTGAA; Reverse: TGCTGGATATCTGCAGAATTCTCAGTCTTTGGCATCTCCCACTC) containing the EcoR I or BamH I restriction site and, subsequently, subcloned into the PCNA3.1 plasmid (Origene, Rockville, MD) to construct the tag-free plasmid. Cells were seeded on 60mm dishes and Rad51 plasmids were transfected 24 h later using Lipofectamine 2000 (Invitrogen, 11668–019), according to the manufacturer’s instructions.

### siRNA transfection

Human p53 siRNA and negative control siRNA(NC) were obtained from GenePharma Co. Ltd. Cells were seeded on 60mm dishes and siRNA were transfected 24 h later using Oligofectamine 2000 (Invitrogen, 12252–011), according to the manufacturer’s instructions.

### Immunofluorescence

Cells plated on glass culture slides were exposed to gefitinib and / or SN-38 for 36 h. Cells were then fixed with 4% paraformaldehyde for 20 minutes and permeabilized with PBS containing 0.1% Triton X-100. After blocking with 4% bovine serum albumin for 30 minutes, cells were incubated with primary γ-H2AX antibodies (1:200 dilution) overnight. Cells were washed with PBS and then incubated with Alexa Fluor 488-conjugated and rhodamine secondary antibodies (Invitrogen) for 1h, respectively. Then cells were stained with DAPI. Fluorescence was observed using an Olympus Fluorview 1000 confocal microscope.

### Measurement of antitumor activity in vivo

HepG2 xenografts were established by subcutaneously injection of HepG2 cells (5 × 10^6^ cells per animal) into 5- to 6-week-old nude mice. When the tumor volume reached a mean group size of about 100 mm^3^, the mice were randomly divided into control and treatment group. Mice in treatment group were treated with irinotecan (1 mg/kg) every 2 days and/ or gefitinib (100 mg/kg) once daily for 30 days. Irinotecan were dissolved in physiologic saline and gefitinib were dissolved in 0.5% CMC-Na. Tumor volume (V) was calculated as follows: V = (length × width^2^) / 2. All procedures were in accordance with the ethical standards of, and the protocols were approved by, the Animal Ethical and Welfare Committee (AEWC), Center for Drug Safety Evaluation and Research, Zhejiang University.

### Statistical analyses

The results are expressed as the mean ± SD of three independent experiments. Statistical significance were analyzed using Student t test and difference were considered statistically significant when P value < 0.05. *, P<0.05; **, P<0.01; ***, P<0.001.

## Results

### Gefitinib synergizes with SN-38 to inhibit proliferation of HCC cells in vitro

**Fig 1:**
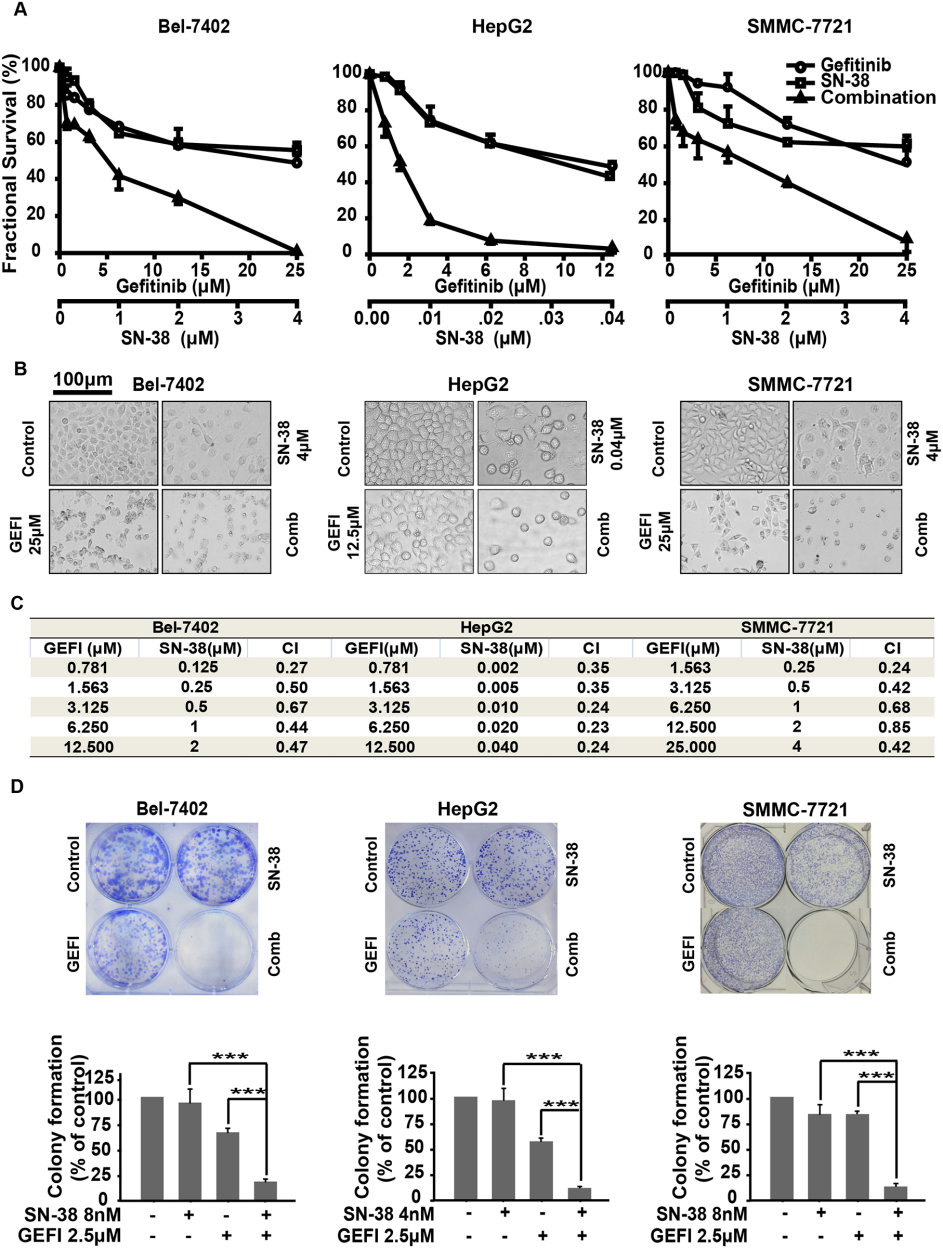
Cytotoxicity of the combination of gefitinib and SN-38. A, Cell proliferation inhibition was examined by the SRB assays. HCC cell lines, including Bel-7402, HepG2 and SMMC-7721 cells were plated in 96-well plates and then exposed to different concentrations of SN-38 and/or gefitinib for 72h. Survival fraction was calculated and shown. B, Cell proliferation inhibition was observed by microscopy. Bel-7402, HepG2 and SMMC-7721 cells were plated in 6-well plates and exposed to indicated dose of gefitinib and/or SN-38 for 72h. Cells were photographed by microscopy. C, CI values at different ratio concentrations of gefitinib and SN-38 were calculated by the proliferation inhibition rates. D, 1,000 Bel-7402, HepG2 and SMMC-7721 cells were plated in a 6-well plate and treated with gefitinib and/or SN-38 for 10days. Then the clonogenic ability was measured and shown. The bars represent the clonogenic ability of different groups. Each study was performed three times and the error bars represent the SD around the mean.

We first used SRB assay to investigate the enhanced effects of the combination of gefitinib and SN-38 on the proliferation of HCC cell lines. Survival curves of gefitinib, SN-38 and combination therapy are shown in Fig 1A. We found that gefitinib or SN-38 alone reduced the viability of Bel-7402, HepG2 and SMMC-7721, while the combination of gefitinib and SN-38 significantly inhibited proliferation. The same results could be observed in six well plate showed in Fig 1B.

To validate the possibility of the combination, we calculated the Combination index (CI) values. As shown in Fig 1C, the combination use of gefitinib and SN-38 had an apparent synergism in Bel-7402, HepG2 and SMMC-7721 cells with CI values <0.8.

Furthermore, a clonogenic assay was performed to assess the long time efficacy of combination treatment on the proliferation and reproductive potential after treatment of HCC cells. We found that treatment with both gefitinib and SN-38 inhibited the colony formation of Bel-7402, HepG2 and SMMC-7721 nearly 100%, even at low concentrations (Fig 1D). Then, we extracted the colonies with 10% acetic acid and measured the absorbance values at 595nm to calculate the quantitative changes of clonogenicity. The combination treatment of gefitinib and SN-38 markedly inhibited colony formation, compared with gefitinib or SN-38 alone. Thus, the synergistic cytotoxicity of gefitinib and SN-38 was quite significant.

### Gefitinib enhances SN-38-triggered caspase-dependent apoptosis in HCC cells

**Fig 2:**
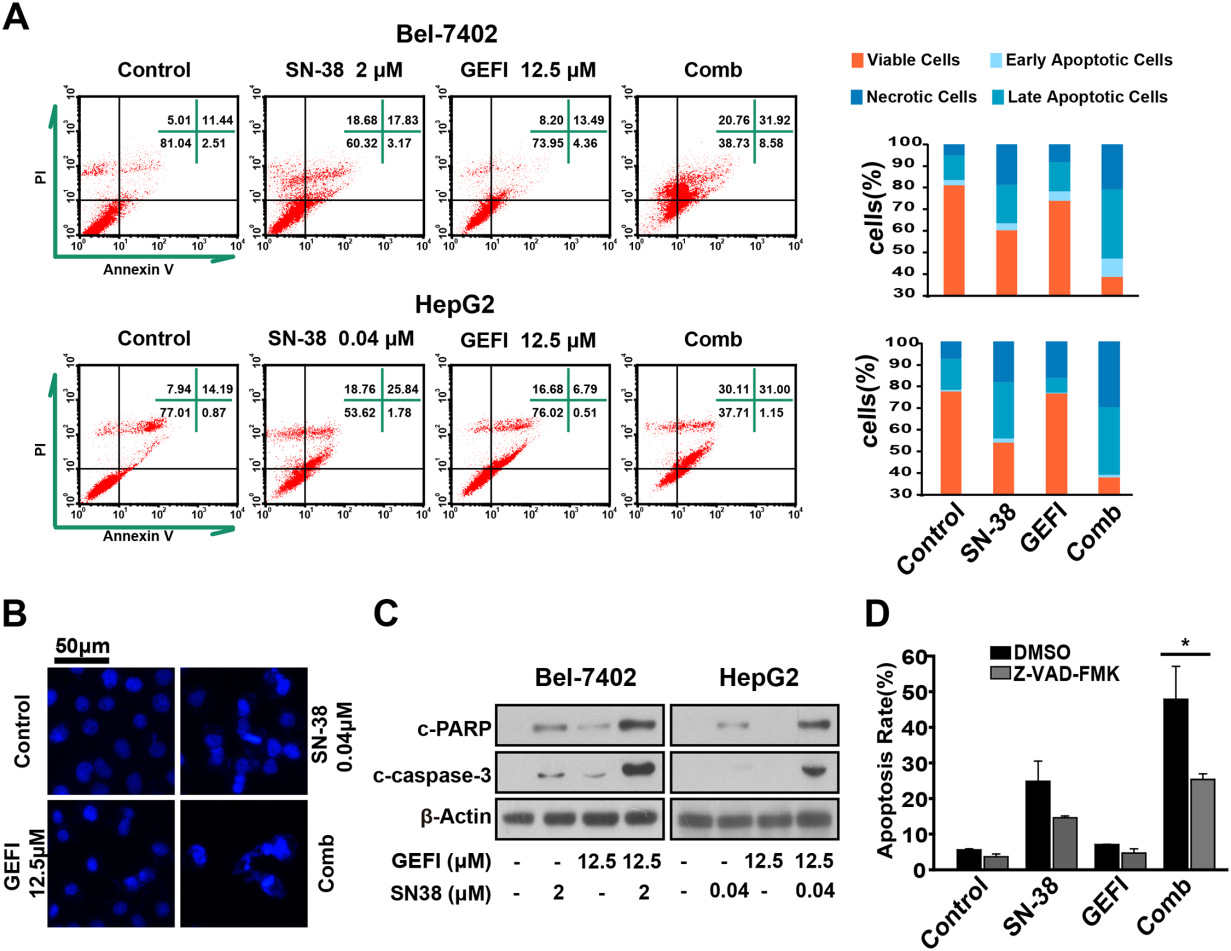
Gefitinib enhances SN-38-triggered apoptosis in HCC cells. A, Bel-7402 and HepG2 cells were exposed to gefitinib and/or SN-38 for 36h. Then, cells were harvested and stained with Annexin V and PI, and apoptosis rates were analysed by flow cytometry and shown. B, HepG2 cells were stained with DAPI after drug treatment as above and nuclear changes were observed on fluorescence microscopy. C, Augmention of apoptosis was proved by western blot analysis of protein extracts of treated cells with specific antibodies against cleaved-PARP, and cleaved-caspase-3. D, HepG2 cells were pretreated with 20μM Z-VAD-FMK (the pan-caspase inhibitor) for 1h and treated with 0.04μM SN-38 and/or 12.5μM gefitinib for 24 h. The cells were then fixed with 70% ethanol at -20°C and stained with PI and analysed on flow cytometry. The experiments were performed three times independently, and the error bars represent the SD around the mean.

Subsequently to further explore mechanisms of enhanced antitumor activity caused by the combination of gefitinib and SN-38, we examined their effects on apoptosis and caspase signaling pathways. The Annexin V-PI staining assay and PI (sub-G_1_) staining assay were employed to detect level of apoptosis (Fig 2A and 2D). After treatment with gefitinib and/or SN-38 for 36h, we found that cells treated with the two agents together experienced 41% and 32% apoptosis in HepG2 and Bel-7402 cells respectively. As shown in Fig 2A, apoptosis rates in cotreatment groups were higher than either gefitinib or SN-38 induced alone. DAPI staining was used to visualize the apoptosis. We observed extensive nuclear condensation and cellular fragmentation in cells treated with gefitinib plus SN-38(Fig 2B).

Furthermore, we examined the effect of gefitinib, SN-38, and their combination on the activation of caspase cascades. Cleaved caspase-3 and cleaved PARP were markers for apoptotic cells [27, 28]. We found that SN-38 had modest effect on PARP, and caspase-3 and gefitinib significantly increased the cleavage of PARP and caspase-3 caused by SN-38 in HepG2 and Bel-7402 cells (Fig 2C). The apoptosis induced by the combination treatment were partially reversed in the presence of the pan-caspase inhibitor Z-VAD-FMK detected by the PI (sub-G1) staining assay (Fig 2D), indicating that the observed cell apoptosis was caspase dependent.

Taken together, our results demonstrated that combination treatment of gefitinib and SN-38 triggered apparent apoptosis via caspase cascades leading to increased cell death.

### The combination therapy resulted in apparent DNA damages

**Fig 3:**
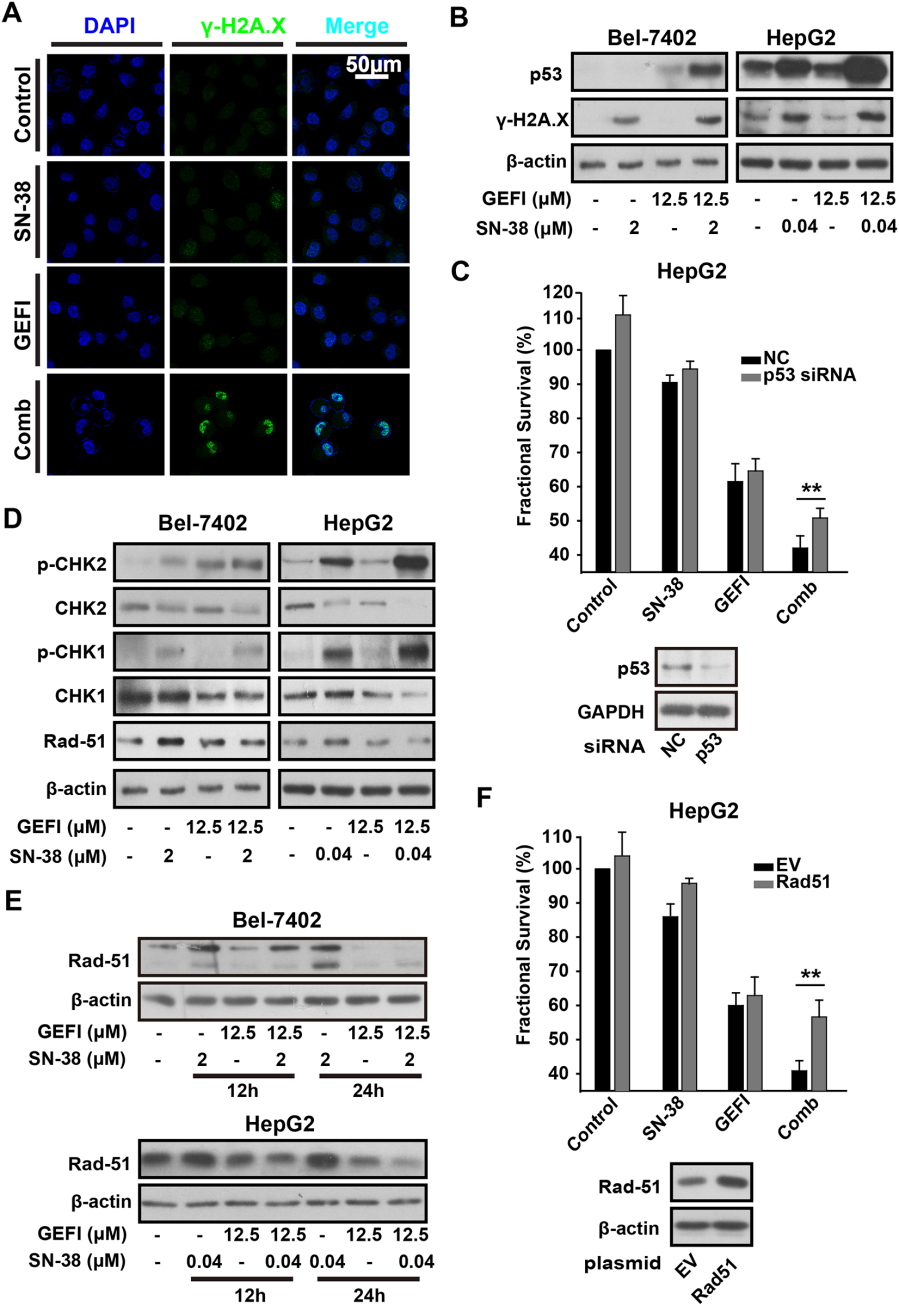
Gefitinib inhibits homologous recombination pathway thus augmenting DNA damges caused by SN-38 in HCC cells. A, HepG2 cells were exposed to gefitinib and/or SN-38 for 24h. γ-H2AX nuclear foci formation was observed by confocal microscopy. B, HepG2 cells were exposed to gefitinib and/or SN-38; Bel-7402 cells were exposed to gefitinib and/or SN-38. Then cells were collected and proteins were extracted. γ-H2AX, p53, p-CHK1, CHK1, p-CHK2, CHK2 and Rad51 protein levels were analysed by Western blot. C, p53 knockdown was achieved by transfection with a p53 siRNA. Western blot analysis with specific antibodies to p53 was used to confirm the effects of p53 knockdown. Then, these cells were treated with 0.04μM SN-38 and/or 12.5μM gefitinib for 36 hours, followed by subsequent proliferation assessment using the SRB assay. D-E, HepG2 and Bel-7402 cells were exposed to gefitinib and/or SN-38; then cells were collected and proteins were extracted. Rad51 protein levels were analysed by western blot. F, 24 hours after transfection with Rad51 expression plasmid or empty vector (PCDNA 3.1, EV), cells were treated with 0.04μM SN-38 and/or 12.5μM gefitinib for 36 hours and proliferation was assessed as mentioned previously.

Previous studies have showed that SN-38 exhibited its antitumor activity by triggering DNA damages. It was hypothesized that increasing DNA damages may be the reason for synergistic activity. Phosphorylation of histone H2AX on serine 139 was one of the earliest cellular responses after the formation of DNA damages. This phosphorylated form of H2AX (referred to as γ-H2AX) was used as a marker for the presence of DNA damages [29]. Here, immunofluorescent assay of γ-H2AX was employed to assess the formation of DNA damages up to this combination. After 6-hour treatment, the drug combination apparently induced γ-H2AX nuclear foci formation, while SN-38 or Gefitinib alone induced little nuclear foci formation(Fig 3A). Increasing phosphorylation of H2AX was also observed using Western blot(Fig 3B). p53 activation was cellular responses to DNA damages, of which protein level is another marker for DNA damages [30]. We found that after 6-hour treatment, the combination of gefitinib and SN-38 apparently induced upregulation of p53 protein(Fig 3B). Totally we proved that the combination therapy of gefitinib and SN-38 resulted in apparent DNA damages. Furthermore, we used siRNA transfection technology to silence p53 and proved that knockdown of p53 in HepG2 cells decrease the combinational effects(Fig 3C). Furthermore, similar results were observed in Hep3B, one of the p53-null HCC cell line, which suggested that the combinational effect was p53 dependent (S1 Fig).

### Accumulation of DNA damages caused by the combination therapy was due to defects of homologous recombination

Homologous recombination (HR) plays a key role in camptothecin-induced DNA damages. Therefore, we speculated that such accumulation of DNA damages was due to defects of HR. Jen-Chung Ko reported that gefitinib could decrease Rad51 stability by promoting 26s proteasome-dependent degradation in human non-small cell lung cancer cells [17, 31]. Consistent with this result, we found that the combination therapy or gefitinib alone led to downregulation of the DNA repair proteins Rad51 in 6 hours (Fig 3D). And other factors involved in HR such as CHK-1 and CHK-2 were phosphorylated after exposure of the drug combination and SN-38 alone. Subsequently, we found that Rad51 was further downregulated after 12 or 24h treatment of combination therapy or gefitinib alone (Fig 3E). Such observation demonstrated that downregulation of Rad51 may be a key reason for the defects of homologous recombination and accumulation of DNA damages. To prove that downregulation of Rad51 is critical for the combinational effects, we applied Rad51 plasmid overexpression assay and demonstrated that overexpression of Rad51 could reverse the combinational effects, as shown in Fig 3F.

### The ubiquitin-proteasome system was involved in gefitinib-induced suppression of Rad51 expression

**Fig 4:**
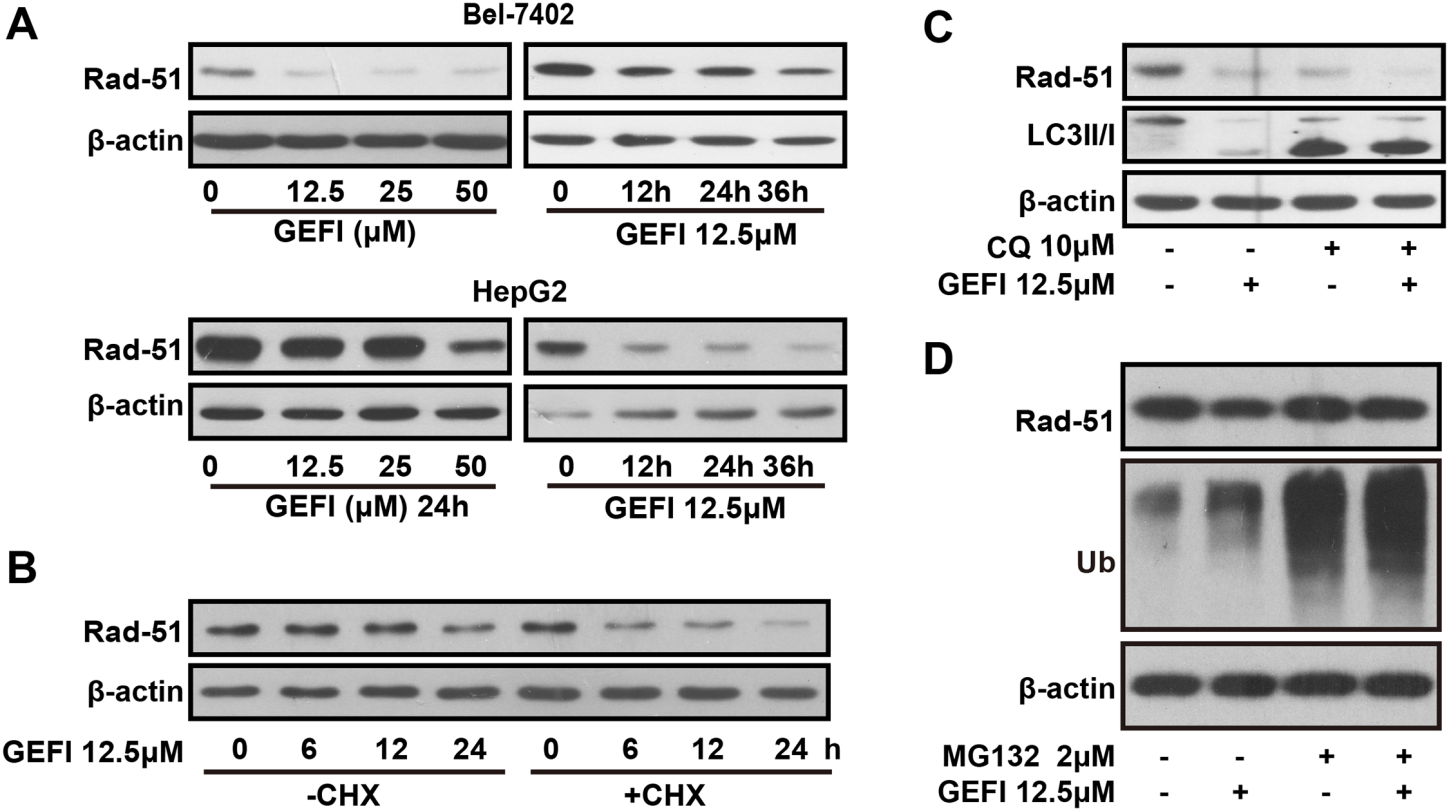
Gefitinib promoted proteasome dependent degradation of Rad51. A and B, Bel-7402 and HepG2 cells were treated with gefitinib. Protein levels of Rad51 were measured by Western blot. C, HepG2 cells were pretreated with CHX for 3 hours or not before exposing to gefitinib (12.5 μM) for different times, and Rad51 protein levels were detected by Western blot. D, HepG2 cells were pretreated with CQ or MG132 for 2 hours and then exposed to gefitinib (12.5 μM) for 24 hours and Rad51 protein levels were measured by western blot.

Results from western blot indicated that gefitinib downregulated Rad51 in a both dose and time dependent manner in Bel-7402 and HepG2 cells (Fig 4A). An inhibitor of protein synthesis CHX was applied to prevent synthesis of Rad51 in HepG2 cells as previous described [32]. We found that CHX pre-treatment could not block the reduction of Rad51, as shown in Fig 4B, which indicated that gefitinib decreased Rad51 protein levels primarily via promoting its degradation. The protein degradation pathways include the ubiquitin-proteasome system and autophagy-lysosome system [33]. HepG2 cells were incubated with lysosomal inhibitors chloroquine (CQ) and proteasome inhibitors MG132 before gefitinib treatment. While CQ had no effect on the gefitinib induced suppression of Rad51, MG132 apparently reversed such reduction, indicating that Rad51 might be degraded through the ubiquitin-proteasome system (Fig 4C and 4D). These findings suggested that gefitinib-induced reductions of Rad51 protein levels might be mediated by the ubiquitin -proteasome pathway.

### Synergistic antitumor efficacy of gefitinib and irinotecan in HepG2 xenografts

**Fig 5:**
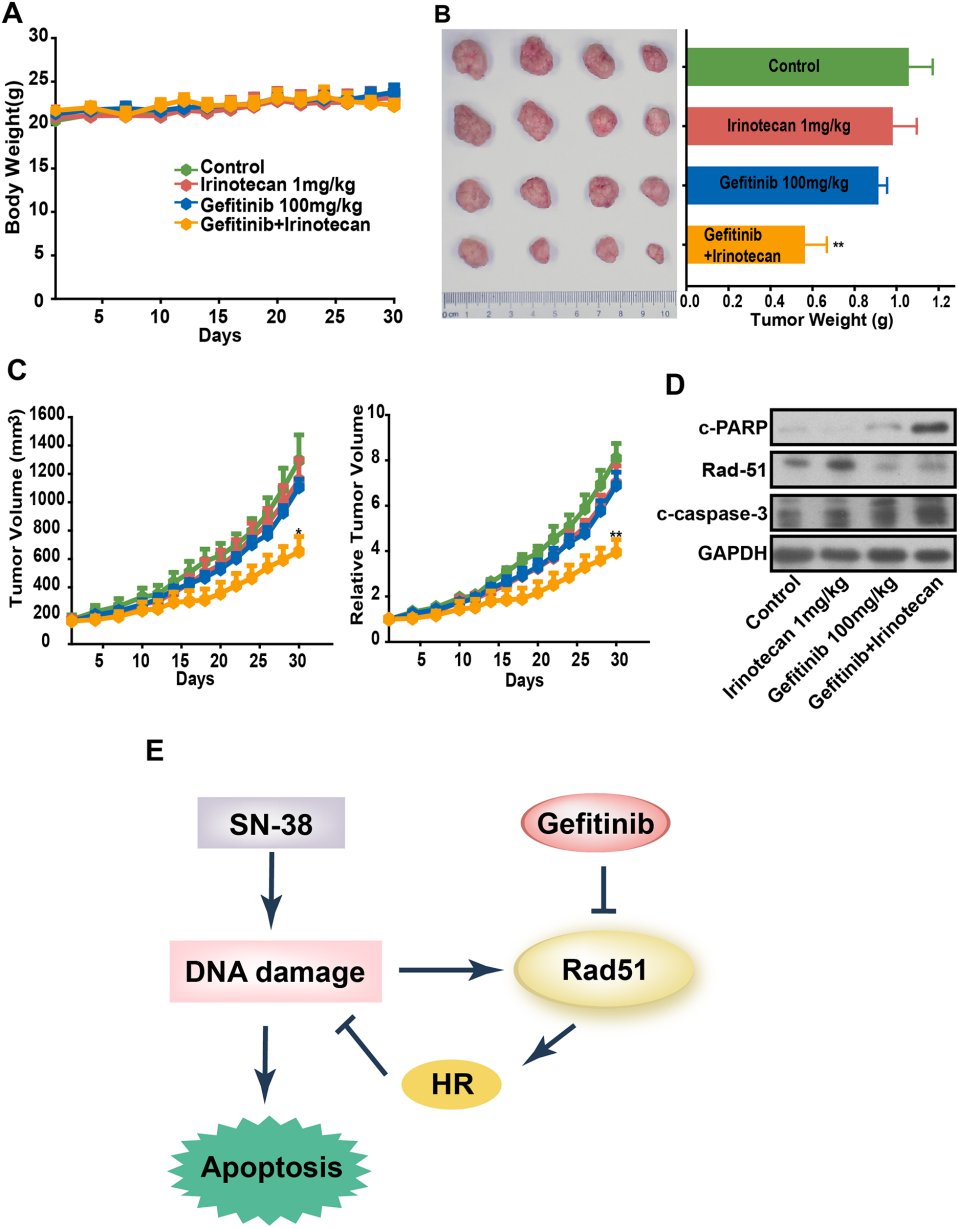
Combination use of irinotecan and gefitinib showed synergistic antitumor effects on HepG2 xenograft models. A to D, Nude mice bearing established HepG2 tumors were treated with irinotecan at 1 mg/kg every 2 days by intraperitoneal injections, and/or gefitinib at 100 mg/kg daily by intragastric administration for 31 days. A, Average body weights were shown as the mean ± SEM. B, Morphology and weight of tumor were expressed as the mean ± SEM. **P < 0.01 versus vehicle treated controls (n = 6/group). C, Tumor volume and relative tumor volume were expressed as the mean ± SEM. D, Protein were extracted from tumor tissues from mice in 4 groups and cleaved-PARP, cleaved caspase-3, cleaved caspase-8 and Rad51 were determined by Western blot. E, model picture to summarize the motif.

To further confirm the synergistic antitumor efficacy of the combination therapy, we tested the in vivo efficacy against HepG2 xenograft model. Irinotecan was administered i.p. every 2 days at 1 mg/kg and gefitinib was administered i.g. at 100mg/kg once daily. During the experiments, no animal death and significant body weight change were observed in all treatment groups(Fig 5A). Besides, we examined the combinational effect in normal hepatocyte cell lines, HL7702, which was no significant cytotoxicity and the results were shown in S2 Fig. This suggested that the combination therapy was tolerated. As shown in Fig 5B, the combination of irinotecan and gefitinib significantly suppressed the tumor growth and such suppression was greater than that caused by gefitinib or irinotecan treatment alone. Furthermore the volume of tumors showed a similar trend to the weight of tumors (Fig 5C). Thus, the combination therapy of gefitinib and irinotecan showed more significant tumor growth inhibitory effects compared with either single agents alone, but caused no observed toxicity; this therapy may be a useful strategy for improving the antitumor activity of irinotecan in HCC.

We next aimed to explore the effect of combination therapy on the expression of apoptosis-related proteins in tumor tissues from xenograft mice. As observed in vitro, Western blot assay showed that caspase cascades were activated in tumor tissue from the nude mice in combination therapy group. Besides, the enhancement of the expression of Rad51 caused by irinotecan was reversed by plusing gefitinib (Fig 5D). In vivo results were consistent with in vitro data, further confirming the synergistic antitumor efficacy of gefitinib and irinotecan was a result of gefitinib aroused defects of HR pathways.

Together, these findings demonstrate that gefitinib promoted proteasome dependent degradation of Rad51 in HCC cell lines, antagonized HR pathway, lead to accumulation of DNA damages and activation of apoptosis, finally resulted in cell death(Fig 5E).

## Discussion

Irinotecan stabilizes cleavable topoisomerase I-DNA complex then results in DNA damages and apoptosis of cancer cells. Clinical studies demonstrated that it fails to exhibit significant effects in patients with hepatocellular carcinoma due to dose-dependent toxicity. Therefore, clinical studies of irinotecan plus other agents aimed at improving its safety and efficacy were paid much attention. It was reported that irinotecan in combination with tyrosine kinase inhibitors(TKIs) such as telatinib and sorafenib caused apparent anti-tumor activity in patients with solid tumors and such strategies was tolerated [34, 35] which indicated that tyrosine kinase inhibitors may synergize with irinotecan to suppress HCC. Thus, to identify the candidate TKI(s) which could improve the safety and efficacy of irinotecan, we performed an in-vitro screening of a TKI library using a SRB assay. We tested the effects of the TKIs in combination with SN38 on the proliferation of HepG2 cells(representative results were showed in S3 Fig). The most potential hit identified by the primary screening was the gefitinib.

Gefitinib is a quinazoline-derived agent that inhibits EGFR-tyrosine kinase. We focused subsequent studies exclusively on gefitinib. CI values suggested that gefitinib plus SN-38 had apparent synergism in HCC cells. Results from colony formation assay further confirmed the enhancement of antitumor activity of SN-38. In addition, we investigated the synergistic effect of gefitinib plus irinotecan using a HepG2 xenograft nude mice model. Body weight monitoring suggested that combination administration of gefitinib and irinotecan had minimal toxicity.

Subsequently, activation of caspase-dependent apoptosis in combination group was observed with Annexin V-PI staining assay and Western blot analyses. In our research we found that reduction of tumor volume and weight and massive tumor cell death were correlated with accumulation of DNA damages detected by the classic DNA damage marker γ-H2AX and p53. Western blots and immunofluorescence assay proved that combination use of SN-38 and gefitinib gave rise to large amount of DNA damages while SN-38 or gefitinib either agents alone induced a little DNA damage. Besides, we proved that knockdown of p53 decrease the combinational effects, which suggested that p53-dependent DNA damage triggered apoptosis is a key factor for inhibition of tumor growth.

Our data above indicated that large amount of DNA damages was caused in combination group. Based on this, we hypothesized that gefitinib block the HR repair pathway to enhance the DNA damage caused by SN-38. We found that CHK1 and CHK2 were phosphorylated after 6 hours exposure to SN-38 plus gefitinib. However, Rad51 protein was decreased in the gefitinib or cotreatment group, and Rad51 plasmid overexpression could reverse the combinational effects, which suggested that gefitinib decreased Rad51 levels to inhibit the HR repair pathway and block of the HR repair pathway was a key reason for the accumulation of DNA damages in HCC cells.

Irinotecan or SN-38 lead to stabilization of covalent topoisomerase I-DNA complexes then cause DNA damages to exhibit their antitumor activity. Such damages get repaired by Rad51-mediated HR repair pathway in mammalian cells. Rad51 is a key DNA repair protein in homologous recombination pathway [14, 36, 37]. After DNA damage, Rad51 concentrates and forms nuclear foci in sites of DNA damage. Jingsong Zhang, et al. reported that E2F1 upregulated Rad51 protein upon DNA damage[38] and Kokichi Sugano demonstrated that Rad51 was recruited upon double-strand DNA breaks induced by SN-38[39] and our results were consistent with these discoveries, indicating that Rad51 might be a therapeutic target. Research showed that deletion of Rad51 gene would sensitize blastocytes of mouse embryos or tumor cells to genotoxic agents [17, 40]. In contrast, overexpression of Rad51 caused more recombination repair and resistance to ionizing radiation or chemotherapeutic agents [41–44]. These reports warrants further (pre)clinical research by concomitant treatment with Rad51 inhibitor and genotoxic agents.

In our study we demonstrated that gefitinib promoted degradation of Rad51 in HCC cell lines. We found that pretreatment with the proteasome inhibitor, MG-132 could reverse gefitinib-induced degradation of Rad51 while autophagy inhibitor CQ could not, which indicated that gefitinib promoted degradation of Rad51 mainly via ubiquitin-proteasome system. Apparently, mechanistic insight into possible mechanisms of this degradation of Rad51 remains to be illuminated for limited studies about Rad51 stability were reported. However, our primary data suggested that gefitinib induced suppression of Rad51 is a promising and clinical feasible strategy to improve the efficacy of DNA-damaging drugs like irinotecan. Meanwhile, our data indicated that ubiquitin-ligating (E3) enzymes for Rad51 may be a potential target for cancer treatment.

In summary, the combination use of topoisomerase I inhibitors and gefitinib exhibited synergistic antitumor activity in vitro and in vivo as proved by decreased tumor cell survival fraction, increased inhibition of tumor cell proliferation, significant activation of apoptosis cascades and increased tumor growth inhibitory rate. Besides, the synergistic activity was related with suppression of Rad51 protein. The combination might be a promising therapeutic strategy for the treatment of HCC which needs to be tested in clinical trials.

## Supporting Information

S1 Fig Synergistic effects of gefitinib and SN-38 were absent in Hep3B cells. A, Cell proliferation inhibition was examined by the SRB assays. Hep3B cells were plated in 96-well plates and then exposed to different concentrations of SN-38 and/or gefitinib for 72h. Survival fraction was calculated and shown.
(TIF)

S2 Fig No significant cytotoxicity of the combinational use of SN-38 and gefitinib to HL7702 was observed. A, Cell proliferation inhibition was examined by the SRB assays. HL7702 cells were plated in 96-well plates and then exposed to different concentrations of SN-38 and/or gefitinib for 72h. Survival fraction was calculated and shown.
(TIF)

S3 Fig Synergistic effects of TKIs and SN-38 in HepG2 cells. A-F, Cell proliferation inhibition was examined by the SRB assays. HepG2 cells were plated in 96-well plates and then exposed to different concentrations of SN-38 and/or TKIs for 48h. Survival fraction was calculated and shown.
(TIF)

## References

[1] Jemal A B F, Center MM, Ferlay J, Ward E, Forman D. Global cancer statistics. CA Cancer J Clin. 2011; 61(2):69–90. 10.3322/caac.20107 21296855

[2] Njei B, Rotman Y, Ditah I, Lim JK. Emerging trends in hepatocellular carcinoma incidence and mortality. Hepatology. 2015;61(1):191–9. Epub 2014/08/22. 10.1002/hep.27388 . 25142309 PMC4823645

[3] Bruix J, Sherman M. Management of hepatocellular carcinoma: an update. Hepatology. 2011;53(3):1020–2. Epub 2011/03/05. 10.1002/hep.24199 21374666 PMC3084991

[4] El-Serag HB, Marrero JA, Rudolph L, Reddy KR. Diagnosis and treatment of hepatocellular carcinoma. Gastroenterology. 2008;134(6):1752–63. 10.1053/j.gastro.2008.02.090 . 18471552

[5] Llovet JM, Bruix J. Novel advancements in the management of hepatocellular carcinoma in 2008. Journal of hepatology. 2008;48 Suppl 1:S20–37. Epub 2008/02/29. 10.1016/j.jhep.2008.01.022 . 18304676

[6] Lu DS, Yu NC, Raman SS, Limanond P, Lassman C, Murray K, et al Radiofrequency ablation of hepatocellular carcinoma: treatment success as defined by histologic examination of the explanted liver. Radiology. 2005;234(3):954–60. Epub 2005/02/01. 10.1148/radiol.2343040153 . 15681691

[7] Llovet JM, Ricci S, Mazzaferro V, Hilgard P, Gane E, Blanc JF, et al Sorafenib in advanced hepatocellular carcinoma. The New England journal of medicine. 2008;359(4):378–90. Epub 2008/07/25. 10.1056/NEJMoa0708857 . 18650514

[8] Fukuoka M, Niitani H, Suzuki A, Motomiya M, Hasegawa K, Nishiwaki Y, et al A phase II study of CPT-11, a new derivative of camptothecin, for previously untreated non-small-cell lung cancer. J Clin Oncol. 1992;10(1):16–20. . 1309380 10.1200/JCO.1992.10.1.16

[9] Masuda N, Fukuoka M, Kusunoki Y, Matsui K, Takifuji N, Kudoh S, et al CPT-11: a new derivative of camptothecin for the treatment of refractory or relapsed small-cell lung cancer. J Clin Oncol. 1992;10(8):1225–9. . 1321891 10.1200/JCO.1992.10.8.1225

[10] Shihabi S, Belani CP. Role of topoisomerase I inhibitors in small-cell lung cancer. Clin Lung Cancer. 2001;2(4):275–81. . 14720360 10.3816/clc.2001.n.010

[11] Saltz LB, Douillard JY, Pirotta N, Alakl M, Gruia G, Awad L, et al Irinotecan plus fluorouracil/leucovorin for metastatic colorectal cancer: a new survival standard. Oncologist. 2001;6(1):81–91. . 11161231 10.1634/theoncologist.6-1-81

[12] Tomicic MT, Kaina B. Topoisomerase degradation, DSB repair, p53 and IAPs in cancer cell resistance to camptothecin-like topoisomerase I inhibitors. Biochim Biophys Acta. 2013;1835(1):11–27. 10.1016/j.bbcan.2012.09.002 . 23006513

[13] Arnaudeau C, Lundin C, Helleday T. DNA double-strand breaks associated with replication forks are predominantly repaired by homologous recombination involving an exchange mechanism in mammalian cells. J Mol Biol. 2001;307(5):1235–45. 10.1006/jmbi.2001.4564 . 11292338

[14] Haaf T, Golub EI, Reddy G, Radding CM, Ward DC. Nuclear foci of mammalian Rad51 recombination protein in somatic cells after DNA damage and its localization in synaptonemal complexes. Proc Natl Acad Sci U S A. 1995;92(6):2298–302. 7892263 10.1073/pnas.92.6.2298 PMC42471

[15] O'Reilly EM, Stuart KE, Sanz-Altamira PM, Schwartz GK, Steger CM, Raeburn L, et al A phase II study of irinotecan in patients with advanced hepatocellular carcinoma. Cancer. 2001;91(1):101–5. . 11148565 10.1002/1097-0142(20010101)91:1<101::aid-cncr13>3.0.co;2-k

[16] Boige V, Taieb J, Hebbar M, Malka D, Debaere T, Hannoun L, et al Irinotecan as first-line chemotherapy in patients with advanced hepatocellular carcinoma: a multicenter phase II study with dose adjustment according to baseline serum bilirubin level. Eur J Cancer. 2006;42(4):456–9. 10.1016/j.ejca.2005.09.034 . 16427779

[17] Ko JC, Ciou SC, Cheng CM, Wang LH, Hong JH, Jheng MY, et al Involvement of Rad51 in cytotoxicity induced by epidermal growth factor receptor inhibitor (gefitinib, IressaR) and chemotherapeutic agents in human lung cancer cells. Carcinogenesis. 2008;29(7):1448–58. 10.1093/carcin/bgn130 . 18544565

[18] Woodburn JR. The epidermal growth factor receptor and its inhibition in cancer therapy. Pharmacol Ther. 1999;82(2–3):241–50. . 10454201 10.1016/s0163-7258(98)00045-x

[19] Ciardiello F, Caputo R, Bianco R, Damiano V, Fontanini G, Cuccato S, et al Inhibition of growth factor production and angiogenesis in human cancer cells by ZD1839 (Iressa), a selective epidermal growth factor receptor tyrosine kinase inhibitor. Clin Cancer Res. 2001;7(5):1459–65. . 11350918

[20] O'Dwyer PJ, Giantonio BJ, Levy DE, Kauh JS, Fitzgerald DB, Benson AB. Gefitinib in advanced unresectable hepatocellular carcinoma: Results from the Eastern Cooperative Oncology Group's Study E1203. J Clin Oncol. 2006;24(18):213s–s. WOS:000239009401288.

[21] Kishida O, Miyazaki Y, Murayama Y, Ogasa M, Miyazaki T, Yamamoto T, et al Gefitinib (Iressa, ZD1839) inhibits SN38-triggered EGF signals and IL-8 production in gastric cancer cells. Cancer Chemother Pharmacol. 2005;55(6):584–94. 10.1007/s00280-004-0959-y . 15723219

[22] Yashiro M, Qiu H, Hasegawa T, Zhang X, Matsuzaki T, Hirakawa K. An EGFR inhibitor enhances the efficacy of SN38, an active metabolite of irinotecan, in SN38-refractory gastric carcinoma cells. Br J Cancer. 2011;105(10):1522–32. 10.1038/bjc.2011.397 21997136 PMC3242520

[23] Vichai V, Kirtikara K. Sulforhodamine B colorimetric assay for cytotoxicity screening. Nat Protoc. 2006;1(3):1112–6. 10.1038/nprot.2006.179 . 17406391

[24] Chou TC, Talalay P. Generalized equations for the analysis of inhibitions of Michaelis-Menten and higher-order kinetic systems with two or more mutually exclusive and nonexclusive inhibitors. Eur J Biochem. 1981;115(1):207–16. . 7227366 10.1111/j.1432-1033.1981.tb06218.x

[25] Chou TC, Talalay P. Quantitative analysis of dose-effect relationships: the combined effects of multiple drugs or enzyme inhibitors. Adv Enzyme Regul. 1984;22:27–55. . 6382953 10.1016/0065-2571(84)90007-4

[26] Chou TC. Drug combination studies and their synergy quantification using the Chou-Talalay method. Cancer Res. 2010;70(2):440–6. 10.1158/0008-5472.CAN-09-1947 . 20068163

[27] Chang HY, Yang X. Proteases for cell suicide: functions and regulation of caspases. Microbiol Mol Biol Rev. 2000;64(4):821–46. 11104820 10.1128/mmbr.64.4.821-846.2000 PMC99015

[28] Oliver FJ, de la Rubia G, Rolli V, Ruiz-Ruiz MC, de Murcia G, Murcia JM. Importance of poly(ADP-ribose) polymerase and its cleavage in apoptosis. Lesson from an uncleavable mutant. J Biol Chem. 1998;273(50):33533–9. . 9837934 10.1074/jbc.273.50.33533

[29] Rogakou EP, Pilch DR, Orr AH, Ivanova VS, Bonner WM. DNA double-stranded breaks induce histone H2AX phosphorylation on serine 139. J Biol Chem. 1998;273(10):5858–68. . 9488723 10.1074/jbc.273.10.5858

[30] Kastan MB, Onyekwere O, Sidransky D, Vogelstein B, Craig RW. Participation of p53 protein in the cellular response to DNA damage. Cancer Res. 1991;51(23 Pt 1):6304–11. . 1933891

[31] Ko JC, Hong JH, Wang LH, Cheng CM, Ciou SC, Lin ST, et al Role of repair protein Rad51 in regulating the response to gefitinib in human non-small cell lung cancer cells. Mol Cancer Ther. 2008;7(11):3632–41. 10.1158/1535-7163.MCT-08-0578 . 19001445

[32] Liu XW, Cai TY, Zhu H, Cao J, Su Y, Hu YZ, et al Q6, a novel hypoxia-targeted drug, regulates hypoxia-inducible factor signaling via an autophagy-dependent mechanism in hepatocellular carcinoma. Autophagy. 2014;10(1):111–22. 10.4161/auto.26838 . 24220190 PMC4389865

[33] Rubinsztein DC. The roles of intracellular protein-degradation pathways in neurodegeneration. Nature. 2006;443(7113):780–6. 10.1038/nature05291 . 17051204

[34] Horiike A, Kudo K, Miyauchi E, Ohyanagi F, Kasahara K, Horai T, et al Phase I study of irinotecan and gefitinib in patients with gefitinib treatment failure for non-small cell lung cancer. Br J Cancer. 2011;105(8):1131–6. 10.1038/bjc.2011.375 21915126 PMC3208500

[35] Langenberg MH, Witteveen PO, Roodhart JM, Verheul HM, Mergui-Roelvink M, van der Sar J, et al Phase I evaluation of telatinib, a vascular endothelial growth factor receptor tyrosine kinase inhibitor, in combination with irinotecan and capecitabine in patients with advanced solid tumors. Clin Cancer Res. 2010;16(7):2187–97. 10.1158/1078-0432.CCR-09-2436 . 20233884

[36] Shinohara A, Ogawa H, Ogawa T. Rad51 Protein Involved in Repair and Recombination in Saccharomyces-Cerevisiae Is a Reca-Like Protein. Cell. 1992;69(3):457–70. 10.1016/0092-8674(92)90447-K WOS:A1992HT07800008. 1581961

[37] Chen JJ, Silver D, Cantor S, Livingston DM, Scully R. BRCA1, BRCA2, and Rad51 operate in a common DNA damage response pathway. Cancer Res. 1999;59(7 Suppl):1752s–6s. . 10197592

[38] Wu M, Wang X, McGregor N, Pienta KJ, Zhang J. Dynamic regulation of Rad51 by E2F1 and p53 in prostate cancer cells upon drug-induced DNA damage under hypoxia. Mol Pharmacol. 2014;85(6):866–76. 10.1124/mol.113.090688 24627085 PMC4014666

[39] Tahara M, Inoue T, Sato F, Miyakura Y, Horie H, Yasuda Y, et al The use of Olaparib (AZD2281) potentiates SN-38 cytotoxicity in colon cancer cells by indirect inhibition of Rad51-mediated repair of DNA double-strand breaks. Mol Cancer Ther. 2014;13(5):1170–80. 10.1158/1535-7163.MCT-13-0683 . 24577941

[40] Tsuzuki T, Fujii Y, Sakumi K, Tominaga Y, Nakao K, Sekiguchi M, et al Targeted disruption of the Rad51 gene leads to lethality in embryonic mice. Proc Natl Acad Sci U S A. 1996;93(13):6236–40. 8692798 10.1073/pnas.93.13.6236 PMC39005

[41] Vispe S, Cazaux C, Lesca C, Defais M. Overexpression of Rad51 protein stimulates homologous recombination and increases resistance of mammalian cells to ionizing radiation. Nucleic Acids Res. 1998;26(12):2859–64. 9611228 10.1093/nar/26.12.2859 PMC147643

[42] Arnaudeau C, Helleday T, Jenssen D. The RAD51 protein supports homologous recombination by an exchange mechanism in mammalian cells. J Mol Biol. 1999;289(5):1231–8. 10.1006/jmbi.1999.2856 . 10373364

[43] Takenaka T, Yoshino I, Kouso H, Ohba T, Yohena T, Osoegawa A, et al Combined evaluation of Rad51 and ERCC1 expressions for sensitivity to platinum agents in non-small cell lung cancer. Int J Cancer. 2007;121(4):895–900. 10.1002/ijc.22738 . 17417781

[44] Schneider S, Park DJ, Yang DY, El-Khoueiry A, Sherrod A, Groshen S, et al Gene expression in tumor-adjacent normal tissue is associated with recurrence in patients with rectal cancer treated with adjuvant chemoradiation. Pharmacogenet Genom. 2006;16(8):555–63. 10.1097/01.fpc.0000220563.44724.6d WOS:000239970700003. 16847424

