# Supplementary figures and images for: Gefitinib Synergizes with Irinotecan to Suppress Hepatocellular Carcinoma via Antagonizing Rad51-Mediated DNA-Repair
Source: PLoS One. 2016 Jan 11;11(1):e0146968. doi: 10.1371/journal.pone.0146968 (PMC4709237; doi:10.1371/journal.pone.0146968)

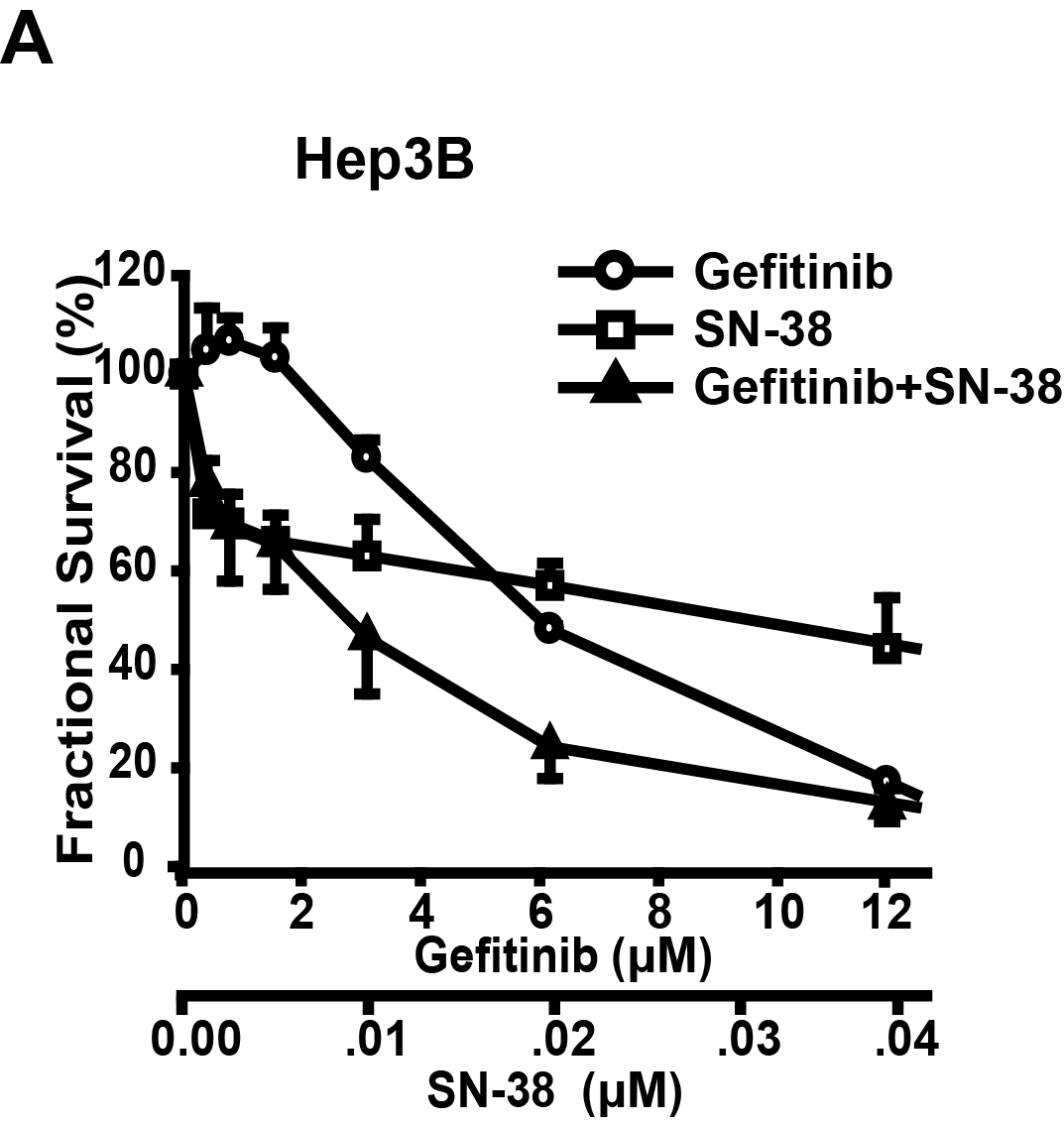

Supplement: S1 Fig — A, Cell proliferation inhibition was examined by the SRB assays. Hep3B cells were plated in 96-well plates and then exposed to different concentrations of SN-38 and/or gefitinib for 72h. Survival fraction was calculated and shown. (TIF) [file pone.0146968.s001.tif]

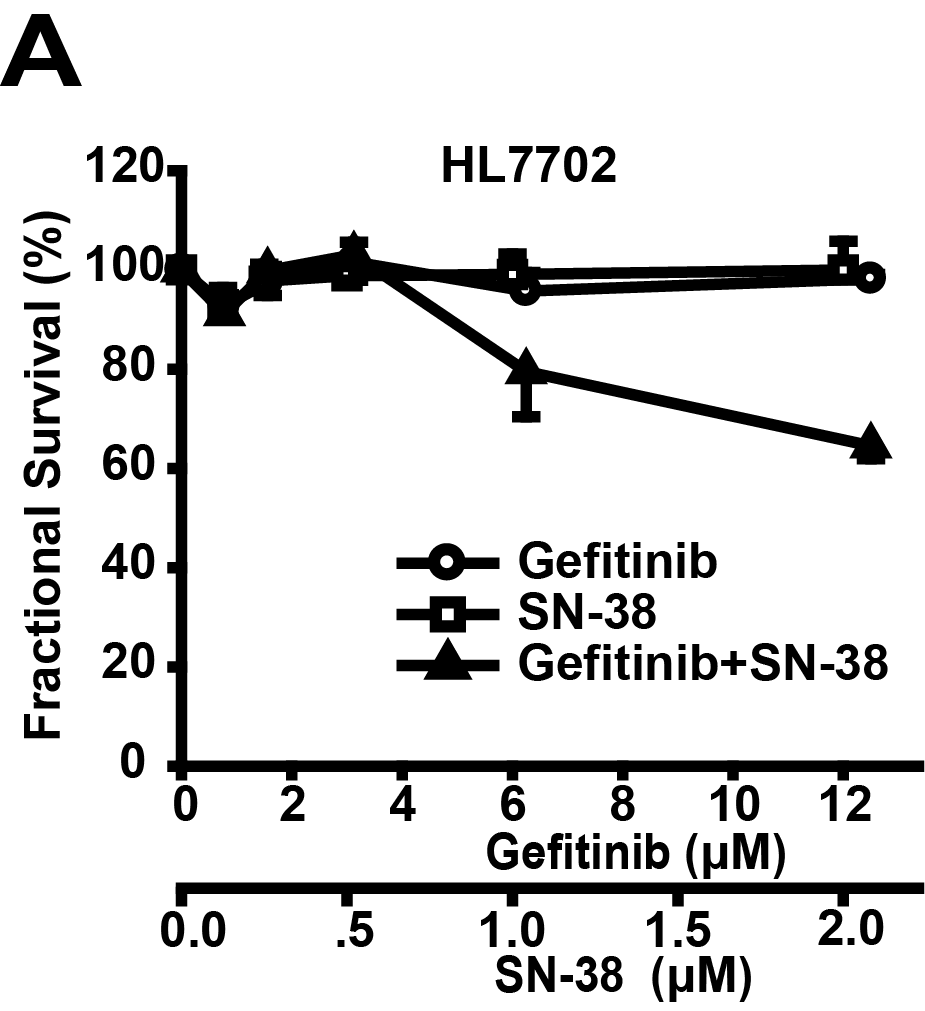

Supplement: S2 Fig — A, Cell proliferation inhibition was examined by the SRB assays. HL7702 cells were plated in 96-well plates and then exposed to different concentrations of SN-38 and/or gefitinib for 72h. Survival fraction was calculated and shown. (TIF) [file pone.0146968.s002.tif]

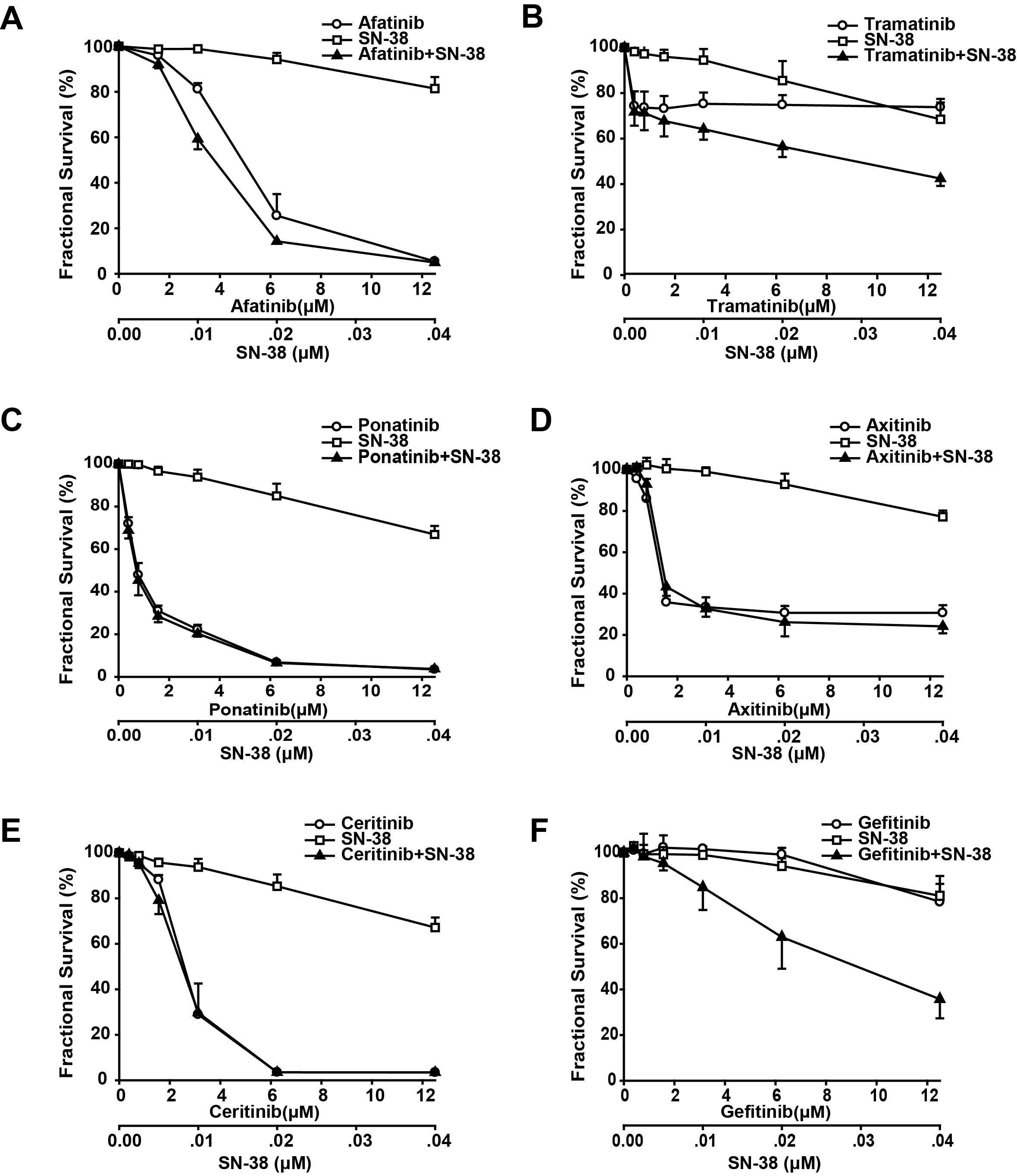

Supplement: S3 Fig — A-F, Cell proliferation inhibition was examined by the SRB assays. HepG2 cells were plated in 96-well plates and then exposed to different concentrations of SN-38 and/or TKIs for 48h. Survival fraction was calculated and shown. (TIF) [file pone.0146968.s003.tif]
